# Validation and promise of a TCR mimic antibody for cancer immunotherapy of hepatocellular carcinoma

**DOI:** 10.1038/s41598-022-15946-5

**Published:** 2022-07-15

**Authors:** Chang Liu, Hong Liu, Moumita Dasgupta, Lance M. Hellman, Xiaogang Zhang, Kai Qu, Hui Xue, Yun Wang, Fenling Fan, Qi Chang, Duo Yu, Linhu Ge, Yu Zhang, Ziyou Cui, Pengbo Zhang, Bradley Heller, Hongbing Zhang, Bingyin Shi, Brian M. Baker, Cheng Liu

**Affiliations:** 1 The First Affiliated Hospital of Xi’an Jiaotong University, Xi’an, Shaanxi Province China; 2 Eureka Therapeutics Inc., 5858 Horton Street, Suite 170, Emeryville, CA USA; 3 Department of Chemistry & Biochemistry and the Harper Cancer Research Institute, University of Notre Dame, 251 Nieuwland Science Hall, Notre Dame, IN USA

**Keywords:** Immunotherapy, Cancer, Structural biology

## Abstract

Monoclonal antibodies are at the vanguard of the most promising cancer treatments. Whereas traditional therapeutic antibodies have been limited to extracellular antigens, T cell receptor mimic (TCRm) antibodies can target intracellular antigens presented by cell surface major histocompatibility complex (MHC) proteins. TCRm antibodies can therefore target a repertoire of otherwise undruggable cancer antigens. However, the consequences of off-target peptide/MHC recognition with engineered T cell therapies are severe, and thus there are significant safety concerns with TCRm antibodies. Here we explored the specificity and safety profile of a new TCRm-based T cell therapy for hepatocellular carcinoma (HCC), a solid tumor for which no effective treatment exists. We targeted an alpha-fetoprotein peptide presented by HLA-A*02 with a highly specific TCRm, which crystallographic structural analysis showed binds directly over the HLA protein and interfaces with the full length of the peptide. We fused the TCRm to the γ and δ subunits of a TCR, producing a signaling AbTCR construct. This was combined with an scFv/CD28 co-stimulatory molecule targeting glypican-3 for increased efficacy towards tumor cells. This AbTCR + co-stimulatory T cell therapy showed potent activity against AFP-positive cancer cell lines in vitro and an in an in vivo model and undetectable activity against AFP-negative cells. In an in-human safety assessment, no significant adverse events or cytokine release syndrome were observed and evidence of efficacy was seen. Remarkably, one patient with metastatic HCC achieved a complete remission after nine months and ultimately qualified for a liver transplant.

## Introduction

Antibody therapeutics have revolutionized oncology due to their ability to mediate destruction of cancer cells and regulate immune cell activity. Such biologics have been raised against and target readily accessible immunogenic epitopes of cell surface proteins. However, fragments of discrete intracellular proteins also exist on the cell surface when presented by class I major histocompatibility complexes (MHC). Recognition of these peptide-MHC (pMHC) complexes by T cell receptors (TCRs) drives cellular immune responses and is associated with naturally occurring and pharmacologically-induced tumor regression. Although TCRs and transgenic T cells have been explored as cancer therapeutics, they have achieved mixed outcomes and are approached with considerable caution^1–6^, due in part to the cross-reactivity characteristic of naturally occurring TCRs that can lead to off-target recognition^7–10^. As an alternative, T cell receptor-mimic (TCRm) antibodies have been developed to bind pMHC complexes, offering the potential to deploy mature antibody engineering methods that can help overcome limitations inherent to TCRs^11,12^.

We previously engineered and described a TCRm that detects an HLA-A*02-presented peptide derived from alpha fetoprotein (AFP)^13^. AFP is a biomarker for hepatocellular carcinoma (HCC), the fourth leading cause of global cancer deaths with a mortality rate of almost 100%^14^. In prior work, this TCRm displayed high specificity and showed no evidence of off-target recognition. Having characterized this AFP TCRm in vitro and in an in vivo model, we now report and characterize an “AbTCR” format in which the heavy and light chains of the F(ab) domain of the AFP TCRm are fused to the constant and transmembrane domains of a γδ TCR^15^.

In addition to the AbTCR we developed a co-stimulatory molecule (co-stim) comprised of an scFv against the cell surface tumor antigen glypican-3 (GPC3) fused to the transmembrane and intracellular domains of CD28. We targeted GPC3 because it has been shown to be overexpressed in 70–100% of HCC tumors^16–20^. The co-stim acts as a coincidence detector that provides anti-anergic signaling when the T cells interact with targets expressing both antigens and helps ensure specific, enduring immune responses against tumor as opposed to healthy cells^21–23^.

We hypothesized that the benefits of the AbTCR + co-stim would make a safe and effective TCRm-based therapy for patients. Indeed, we found that AbTCR + co-stim T cells exhibited improved tumor infiltration and better reduction of HepG2 solid tumors in a mouse model relative to the control. High specificity was retained, which X-ray crystallograhic and biophysical data show emerges from high affinity AbTCR engagement of the AFP/HLA-A*02 complex in a manner that maximizes interactions across the length of the AFP peptide. We used these and other pre-clinical data to proceed to a human study where we observed a clean safety profile and evidence of activity in most of the HCC patients treated. Notably, one advanced patient with metastases in the lung exhibited a CR (complete remission) with few treatment-related adverse events and later qualified for a liver transplant. These findings provide a first-in-class demonstration of the in-human safety of an engineered TCRm platform. They support the progression to further clinical studies to gauge efficacy and provide strong support for additional studies of TCRm molecules as next-generation cancer therapeutics.

## Methods

### Cell lines

Cell lines were obtained from ATCC. To generate SK-HEP-1-MG, the parental SK-HEP-1 line was stably transduced with a pLenti vector carrying a fragment of the AFP gene spanning the peptide region. HepG2-CD80 cells were similarly generated from HepG2 cells by expressing human CD80 cDNA. Healthy human donor peripheral blood leukocytes were obtained from Blood Centers of the Pacific and T cells were isolated using an EasySep Human T Cell Isolation Kit (StemCell Technologies). The HepG2 cells were previously confirmed to lack mutated BRAF^24^.

### Generation of AbTCR + co-stim T cells

The anti-AFP TCRm antibody used to bind the AFP_158–166_ peptide complexed with human leukocyte antigen (HLA)-A*02:01 was described previously^13^. The TCR component of AbTCR is derived from the human TCR γ (UniProtKB: locus TRGC1_HUMAN, accession P0CF51) and δ (UniProtKB: locus TRDC_HUMAN, accession B7Z8K6) chains. The antibody raised against GPC3 was discovered using the E-ALPHA® Phage Display system (Eureka Therapeutics) and fused to the transmembrane and intracellular domains of CD28, with an insertion of a myc tag. AbTCR-encoding and co-stim-encoding cDNAs were cloned into a 3rd generation pCDH lentiviral vector (Systems Biosciences) for delivery into T cells. Primary T cells were isolated using negative selection and stimulated with CD3/CD28 Dynabeads (Thermo Fisher Scientific). After 24 h activated T cells were transduced with lentivirus at an MOI of 2–5, and cultured in RPMI with 10% FBS, IL-7 (10 ng/ml), and IL-15 (5 ng/ml) as previously described^13^. Transduction efficiency for receptor positive T cells was determined by flow cytometry using an anti-myc antibody (Cell Signaling) to detect the co-stim or an anti-F(ab’)_2_ antibody (Jackson ImmunoResearch) to detect AbTCR. The percent of receptor positive T cells were normalized with donor-matched non-transduced (mock) T cells for all experiments directly comparing AbTCR with and without co-stim. All comparative results were observed with no fewer than three donors. The sequences of the 20 HLA-A*02 presented, non-AFP related control peptides used are: LLDVPTAAV, TLWVDPYEV, FLLDHLKRV, LLLDVPTAAV, VLFRGGPRGLLAV, SLLPAIVEL, YLLPAIVHI, FLLPTGAEA, LLDPKLCYLL, SLPHFHHPET, MLLSVPLLLG, LLYDMVCGDIP, LLLDVPTAAVQ, LLLDVPTAAVQA, VLFRGGPRGLLAVA, MVDGTLLLL, YMAPEILMRS, FIYNADLMNC, KVNVDEGGE, KQYESVLMVSI.

### Antibodies and recombinant proteins

Primary Antibodies for FACS and immunofluoresence were obtained from BioLegend unless noted otherwise: CD69 (clone FN50), CD107a (clone H4A3), CD3ε (clone UCHT1), PD1 (clone EH12.2H7), TIM3 (clone F38-2E2 ), LAG3 (clone 11C3C65), CCR7 (clone G043H7), CD45RA (clone HI100), CD4 (clone OKT4), CD8 (clone RPA-T8), myc mAb (Cell Signaling Technology, clones 9B11 and 71D10), a goat pAb F(ab)2 (Jackson ImmunoResearch), anti-CD3 epsilon (Abcam # ab52959), a mouse anti-idiotype antibody that detects the AbTCR (Eureka Therapeutics), p-Zap-70 (Cell Signaling #2701) and CD45 (Biolegend, clone HI30). Secondary antibodies were obtained from Jackson ImmunoResearch: Goat anti-rabbit AlexaFluor 555, goat anti-mouse AlexaFluor 488, goat anti-mouse AlexaFluor 647 and biotinylated Goat Anti-Rabbit IgG H&L (ab6720, Abcam). Recombinant TGFbeta was obtained from Peprotech.

### Immunofluorescence

The fixable CMAC dye CellTracker Blue (ThermoFisher) was applied in some experiments to label live target cells before engagement with T cells. AbTCR + co-stim T-cells and target cells were mixed in equal proportions at high density (4 × 10^6^/mL) in complete growth media, centrifuged at 40 × g for 5 min and incubated at room temperature for 30 min to promote synaptogenesis^25^. Cells were gently re-suspended in the same media and 50 µL (2 × 10^5^ cells) were applied to poly-lysine coated glass coverslips and allowed to adhere for 15 min at room temperature. Adhered cells were then fixed and quenched as described previously^26^. PBS with 3% BSA, 1% normal goat serum (Jackson ImmunoResearch) 1% cold water fish gelatin (Sigma Aldrich) and 0.03% TX100 (Sigma Aldrich) was used for all blocking and subsequent antibody incubation steps. Primary antibodies were applied overnight in a humidification chamber at 4 °C and secondary antibodies were applied for 90 min at room temperature. Stained cells were mounted on slides with ProLong Gold antifade reagent (ThermoFisher). Microscopy was carried out on an Olympus IX73 with a 60X UPlanSApo objective (NA 1.35) and images were collected with a Hamamatsu camera C11440 using HCImageLive v4.3 software and processed with Photoshop CC2019.

### Animal studies

Animal experiments were conducted at Lumigenics LLC (750 Alfred Nobel Drive, Suite 103, Hercules, CA). The experimental protocols were approved by the Institutional Animal Care and Use Committee (IACUC) of Lumigenics; animal experiments were conducted and reported in accordance with IACUC and ARRIVE guidelines. Female NSG aged 6–8 weeks were used, with 6 mice per group. HepG2 cells were mixed with 50% Matrigel prior to implantation (5 × 10^6^ cells/mouse) and tumors were measured by calipers. When tumors reached approximately 200 mm^3^, mice were randomized to treatment groups and 5.0 × 10^6^ receptor-positive T cells were intravenously administered in each mouse. 7 days after T cell dosing, a subset of mice was sacrificed for blood analysis and the remaining mice were sacrificed at 21 days. The maximum tumor size allowed for the study was 2500 mm^3^.

### Immunohistochemistry

The tumor tissues were collected and fixed in 10% formalin. The tumor samples were then dehydrated in ethanol and embedded in paraffin. Paraffin sections of the tumor samples (5 um) were cut and IHC was performed as following. Briefly, the paraffin slides were baked at 54–56 °C for 30 min and then went through the deparaffinization steps. Citric acid-based antigen unmasking solution (Vector laboratories, H-3300-250) were used for antigen retrieval. The slides were then blocked with 3% H_2_O_2_ followed by a casein blocking solution. Rabbit anti-CD3 primary antibody was added and incubated for 2 h at room temperature. After washing with PBST, an HRP-conjugated secondary antibody was added and incubated for 30 min. ABC kits from VECTASTAIN (Vector laboratories: PK-6100) were used to amplify the signal. Finally, the DAB solution was added (Vector laboratories: Cat: SK-4105), followed by counter-staining with hematoxylin.

### Generation of recombinant proteins

Soluble, recombinant AFP/HLA-A*02 was generated from bacterially produced inclusion bodies as previously described^27^. Briefly, inclusion bodies of the HLA heavy chain and β_2_-microglobulin, expressed separately in *Escherichia coli*, were purified and denatured in 8 M urea. A 1:3 molar ratio of heavy chain and β_2_-microglobulin along with excess peptide were diluted into refolding buffer containing 100 mM Tris·HCl (pH 8.3), 400 mM ʟ-arginine, 6.3 mM cysteamine, 3.7 mM cystamine, 2 mM EDTA, and 0.2 mM PMSF and were incubated in refolding buffer overnight at 4 °C followed by dialysis against 10 mM Tris·HCl (pH 8.3) for 48 h. Refolded AFP/HLA-A*02 was purified using anion-exchange followed by size-exclusion chromatography. The AFP peptide was purchased from Genscript at > 90% purity. A stock concentration of 30 mM was prepared by dissolving the required amount of the peptide in DMSO for subsequent generation of AFP/HLA-A*02 complexes.

The TCRm Fab fragment was produced from intact human IgG1 formatted antibody (produced by CHO cells) and purified with the Pierce Fab Preparation Kit. Briefly, the human TCRm IgG1 was concentrated (~ 12 mg/mL) in digestion buffer (20 mM sodium phosphate, 10 mM EDTA, 20 mM cysteine-HCl; pH 7.0). Immobilized papain was equilibrated in the digestion buffer (0.5 mL per 10 mg IgG) and added to the antibody. The mixture was shaken at 37 °C for four hours. The resin was separated from the digest, and flow-through fraction contains Fab fragments was purified by an Immobilized Protein A column. The Fab fragments in the flow-through fraction were then further purified by size-exclusion chromatography.

### Protein crystallization, data collection, structure refinement, and analysis

For crystallization of the TCRm-AFP/HLA-A*02 complex, purified TCRm and AFP/HLA-A*02 were mixed at a 1:1 ratio and incubated for 30 min at 4 °C. The incubated mixture was further purified and chromatographically buffer exchanged into 10 mM HEPES and 20 mM NaCl at pH 7.4. Crystals of the TCRm-AFP/HLA-A*02 complex were grown in 18% v/v PEG 3350 and 0.2 M sodium formate, pH 7.2, at 23 °C. For crystallization of the free unbound forms of the TCRm and AFP/HLA-A*02, purified protein was buffer exchanged in 10 mM HEPES and 20 mM NaCl buffer at pH 7.4. Crystals of the free TCRm were grown in 15% w/v polyethylene glycol 6000 and 5% w/v glycerol at 23 °C. Crystals of AFP/HLA-A*02 were grown in 25 mM MES pH 6.5, 150 mM NaCl, 25% v/v PEG 3350 at 4 °C. Total protein concentration for all three crystals was approximately 7 mg/mL. Crystals were grown by hanging drop vapor diffusion using a Mosquito crystallization robot. During harvesting, crystals were soaked in 15% PEG 400/85% mother liquor for cryoprotection and immediately frozen in liquid nitrogen. Diffraction data for the TCRm-AFP/HLA-A*02 complex and the free TCRm were collected at the 24ID-E (NE-CAT) beamline at the Advanced Photon Source, Argonne National Laboratories. Diffraction data for free AFP/HLA-A*02 were collected at the 24ID-C (NE-CAT) beamline.

Indexing, integration and scaling of the data were performed using HKL2000 and DIALS. The TCRm-AFP/HLA-A*02 complex was solved by molecular replacement with Phaser^28^ using a partial solution obtained through MoRDa^29^ for the TCRm with the CDR loops removed and the coordinates of PDB ID 5E00 for the HLA-A*02 with the peptide removed as separate search models^30^. The same models were used for molecular replacement of the unbound free proteins. Molecular replacement was followed by rigid body refinement in PHENIX^31^. AutoBuild was used for model building, followed by multiple restrained refinements with weight optimization using PHENIX Refine^32,33^. Coot was used to analyze the refined models as well as manually fit certain parts of model into the electron density map^34^. MolProbity was used to evaluate structures after each cycle of refinement^35^. Simulated annealing composite OMIT maps were calculated in PHENIX to verify the atomic positions.

All three structures had two molecules per asymmetric unit (AU). The multiple copies were very similar: the variable domains, peptide, and HLA-A*02 peptide binding domain of the TCRm-AFP/HLA-A*02 complex superimposed with a Cα root mean square (RMS) deviation of 0.9 Å; the peptide and peptide binding domains of the AFP/HLA-A*02 complex superimposed with a Cα RMS deviation of 0.2 Å; and the variable domains of the free TCRm superimposed with a RMS deviation of 0.7 Å. All analyses shown here were thus performed for the first molecule in each AU (chains H, L, A, B, C for the TCRm-AFP/HLA-A*02 complex; chains A, B, C for the AFP-HLA-A*02 complex; and chains H, L for the free TCRm). Note that the second molecule in the AU for the TCRm-AFP/HLA-A*02 complex can be generated via a crystallographic symmetry operation. PyMOL and Discovery Studio were used for structural analyses and generating figures. Interatomic contacts were tabulated using ContPro^36^. The TCRm crossing angle was calculated as described previously^37^. The PDB IDs of the structures of the TCRm-AFP/HLA-A*02 complex, the free AFP/HLA-A*02, and free TCRm are 7RE7, 7RE8, and 7RE9.

### Surface plasmon resonance

Kinetic titration experiments were performed using a Biacore T200 instrument using Series S CM5 sensor chips as previously described^38^. Briefly, AFP/HLA-A*02 was amine coupled to the sensor surface to a density of 100–200 response units and a series of five injections of increasing concentrations of purified TCRm were performed at a flow rate of 100 μL/min at 25 °C. Reference and buffer double-subtracted response data were fit with a 1:1 binding model using BIAevaluation 3.0. The reported *k*_on_, *k*_off_, and *K*_D_ values are the mean and standard deviation of three independent experiments.

### Clinical studies

Human safety studies were approved by The Ethics Committee of the First Affiliated Hospital of Xi’an Jiaotong University; studies were performed in accordance with the relevant guidelines and regulations. Informed written consent was obtained from all patients. Two follow-on clinical trials with AbTCR + co-stim T cells using the same antibody domains as those used in the present study have also been registered with US Clinical Trial Database, ClinicalTrials.gov under accession numbers NCT04502082, NCT04634357.

## Results

### TCRm-based T cell therapy is specific and effective in vitro and in an in vivo model

**Figure 1:**
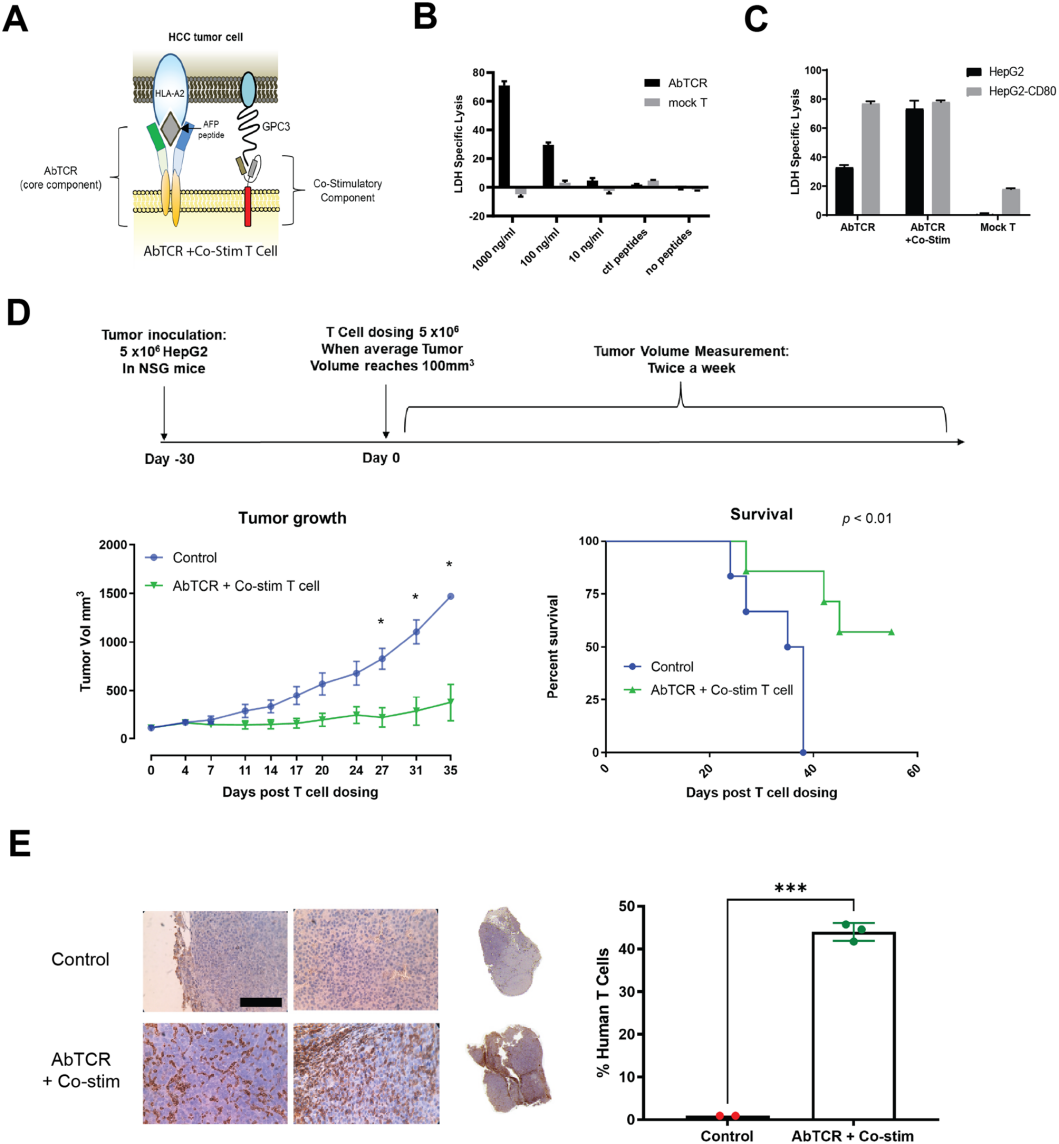
AbTCR + co-stim T cells mediate antigen-specific responses in vitro and in vivo. (**A**) Schematic design of the AbTCR + co-stim T-cell. LDH release was measured as a proxy for T cell-mediated target cell lysis in peptide-loaded T2 cells (**B**) and HepG2 cells (**C**). A total of 20 HLA-A*02 presented, non-AFP related peptides (sequences in Methods) were used as control peptides (ctl peptides in B). (**D**) NSG mice were inoculated with HepG2 cells and treated intravenously with one dose of 5 × 10^6^ T cells when tumors reached approximately 200 mm^3^, using the AbTCR + co-stim as an experimental arm and a CD19-targeting construct as control arms (n = 6 NSG mice/group). Efficacy was measured as the average volume of HepG2 subcutaneous tumors and the rate of animal survival. (**E**) Tumor infiltrating lymphocytes (TILs) in HepG2 subcutaneous tumors were analyzed by immunohistochemical staining for human CD3. Representative images are shown for one tumor of each group with two areas at 40 × magnification (left) and 1 × whole-mount (right). Scale bar in 40x, 100 μm. Quantification of human T cells in the tumors was performed by counting CD3 positive cells and total cells in different areas throughout the section. Each data point represents one tumor sample. Statistical significance indicated as *, *p* < 0.05; ***, *p* < 0.001; ****, *p* < 0.0001.

The AbTCR + co-stim platform was designed to replicate the processes of TCR activation and co-stimulation that naturally drive T cell expansion and cytotoxicity (Fig. 1A). We first observed that this AbTCR + co-stim platform appears to reconstitute synapse formation at the T cell surface (Fig. S1), and thus expanded our investigation to characterize its activity, specificity, and safety profile. We began by examining the killing activity of the T cells expressing TCRm AbTCR alone against T2 cells which express HLA-A*02 but not the TAP transporter, designed to enable custom peptide loading^39^ (Fig. 1B). We found that the AbTCR enables T cells to kill T2 cells loaded with AFP peptide in a dose-dependent manner. In contrast, the AbTCR does not confer killing activity when T2 cells are either unloaded or loaded with a mixture of control peptides. These results suggest that the T cells expressing AbTCR are both effective and specific for killing target cells with the AFP peptide presented in the HLA-A*02 complexes.

To determine if the anti-GPC3 co-stim could boost anti-AFP AbTCR-T cell activity, we performed a killing assay on HepG2 cells as they express HLA-A*02, AFP and GPC3 (Fig. 1C). As a positive control we included HepG2 cells overexpressing CD80, the endogenous co-stimulatory ligand for CD28. The results with the AbTCR + co-stim T cells show that GPC3 can boost killing activity via co-stim as effectively as CD80 via endogenous CD28 (Fig. 1C). The co-stim also performed similarly to CD80 in terms of stimulating host T cell proliferation, cytokine release, and expansion of the central memory compartment (Fig. S2). These data demonstrate that physiological levels of AFP and GPC3 are sufficient to trigger T cell signaling. We then investigated the specificity of AbTCR + co-stim against a panel of cell lines (Fig. S3). Only those lines that expressed both AFP and HLA-A*02 elicited release of IFN-γ and degranulation by AbTCR + co-stim T cells, indicating on-target/on-tumor activity, without evidence of off-target reactivity. This experiment also confirmed that co-stim signaling alone is insufficient to mediate AbTCR + co-stim T cell killing activity.

We next sought to determine the efficacy of the AbTCR + co-stim T cells in vivo. We dosed NSG mice with 5 million HepG2 cells and infused them with the same number of receptor-positive T cells when their tumors reached 100 mm^3^ 30 days later (Fig. 1D). As a negative control we used a CD19-targeting AbTCR ^15^. The results show that the AbTCR + co-stim T cells suppress tumor growth and enhance survival relative to the control arm. Furthermore, we found significant tumor infiltration of the T cells in the AbTCR + co-stim arm (Fig. 1E). Importantly, we did not observe any adverse responses (e.g., labored respiration, slow to responses or movements, etc.) or body weight changes in treated mice, underscoring the safety of the AbTCR + co-stim T cell therapy according to the applicable health indicators. Altogether, these results show that the AbTCR + co-stim T cells have significant activity and high specificity in this HCC animal model.

### The crystallographic structure of the TCRm-AFP/HLA-A*02 complex illuminates the mechanism of TCRm high specificity

**Figure 2:**
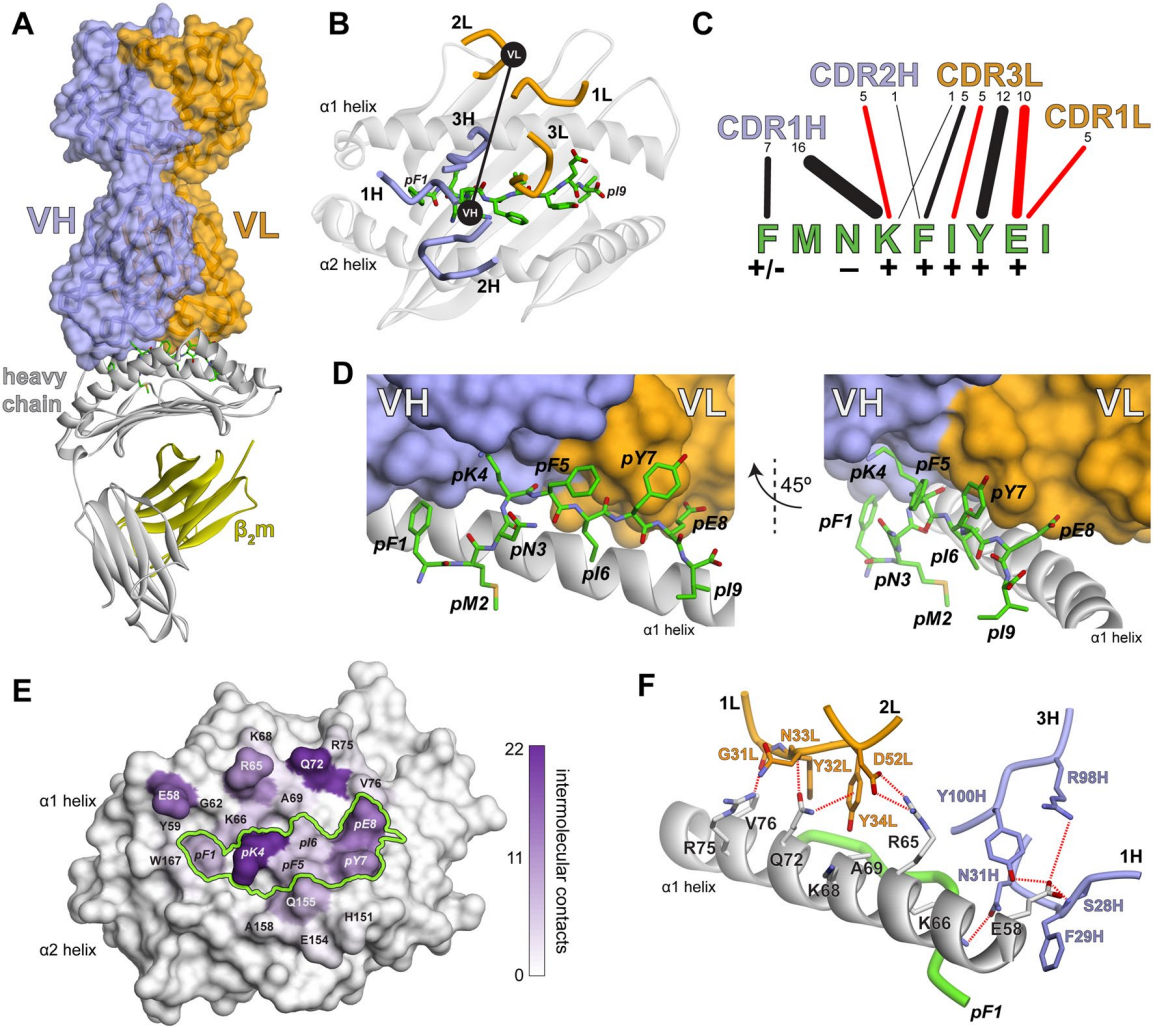
The TCRm binds centrally over the AFP/HLA-A*02 complex engaging the full length of the AFP peptide. (**A**) Overview of the TCRm-AFP/HLA-A*02 complex with the heavy and light chains of the TCRm, the HLA-A*02 heavy chain, peptide, and β_2_-microglobulin colored as indicated. This color scheme is maintained throughout the figure. (**B**) The TCRm and its CDR loops are positioned centrally over AFP/HLA-A*02, forming a diagonal binding mode mimicking that formed by naturally occurring αβ TCRs. The CDR loops are indicated, and black circles indicate the centers of mass of the VH and VL domains. (**C**) TCRm contacts to the peptide are formed by CDR1H, CDR2H, CDR3L, and CDR1L. Contacts from each loop to each peptide amino acid are indicated by lines. The number of contacts is shown by the numbers across the top and represented by line width. Red lines indicate the presence of one or more hydrogen bond or salt bridge. The impact of alanine substitutions at the various peptide positions on TCRm recognition, taken from Ref.^13^, is indicated by the symbols underneath the peptide amino acids. (**D**) Illustration of how the TCRm packs against the peptide, reflecting engagement across the peptide length. (**E**) TCRm contacts to AFP/HLA-A*02 mapped to the surface of the molecule. The number of contacts to each amino acid is indicated by purple-white color scale. The peptide surface is outlined by the green line. Substantially more contacts are made to the HLA-A*02 α1 helix compared to the α2 helix. (**F**) The TCRm interacts with the HLA-A*02 α1 helix via a series of interdigitating side chains that form multiple electrostatic interactions with polymorphic sites as indicated by the dashed red lines.

To illuminate the basis for the high specificity revealed by the in vitro and in vivo assays, we determined the TCRm-AFP/HLA-A*02 crystallographic structure to a resolution of 2.6 Å (Table S1; Fig. S4). Unlike some other instances using TCRm antibodies^40^, the TCRm bound with a clear αβ TCR-like geometry, sitting diagonally over the center of the AFP/HLA-A*02 complex with a TCR-like crossing angle of 48° (Fig. 2A, B)^41^. Strikingly, and unusual for TCRs though, the TCRm interfaced with the peptide along its entire length, using CDR1H, CDR2H, CDR1L, and CDR3L to form significant numbers (≥ 5) of contacts to every residue except for the primary anchors and the asparagine at position 3, which packs against the HLA-A*02 α2 helix (Fig. 2C, D). TCRm engagement across the peptide helps explain high peptide specificity and is well correlated with the significant impact of alanine mutations at each peptide position (Fig. 2C)^13^. Also unusual compared to natural TCRs^42^, the majority of the TCRm contacts to HLA-A*02 were from the CDR1 loops of both chains (Fig. S5). The α1 helix played a much larger role in the interface than α2 (Fig. 2E), and contacts here involved atypical interdigitating side chains and networks of electrostatic interactions with polymorphic HLA residues (Fig. 2F). The atypical contacts likely reflect the use of antibody rather than TCR loops to engage HLA-A*02. This further contributes to specificity, as genes encoding TCR but not antibody CDR loops are believed to have co-evolved alongside MHC genes, contributing to MHC restriction and a built-in propensity for TCR cross-reactivity^10^. The large contact surface and presence of electrostatic networks are correlated with the high affinity of the TCRm to AFP/HLA-A*02, determined here to be 7 nM by surface plasmon resonance (Fig. S6).

We also determined the crystallographic structures of the unbound TCRm and AFP/HLA-A*02 complex (Table S1; Fig. S4). Although the resolutions of these structures were lower than that of the complex, electron density was clear enough to show that, in contrast with many TCR-pMHC interactions^43^, there were no large conformational changes seen in either molecule outside of changes in side chain rotamers, small rigid body shifts of the TCRm variable domains, and slight remodeling of CDR1H (Fig. S7). The rigid interaction between TCRm and AFP/HLA-A*02 likely further contributes to high specificity and affinity, as more flexible interactions between TCRs and their ligands have been associated with higher degrees of cross-reactivity^43,44^.

### TCRm-based T cell therapy showed an excellent safety profile and indications of efficacy in patients

**Figure 3:**
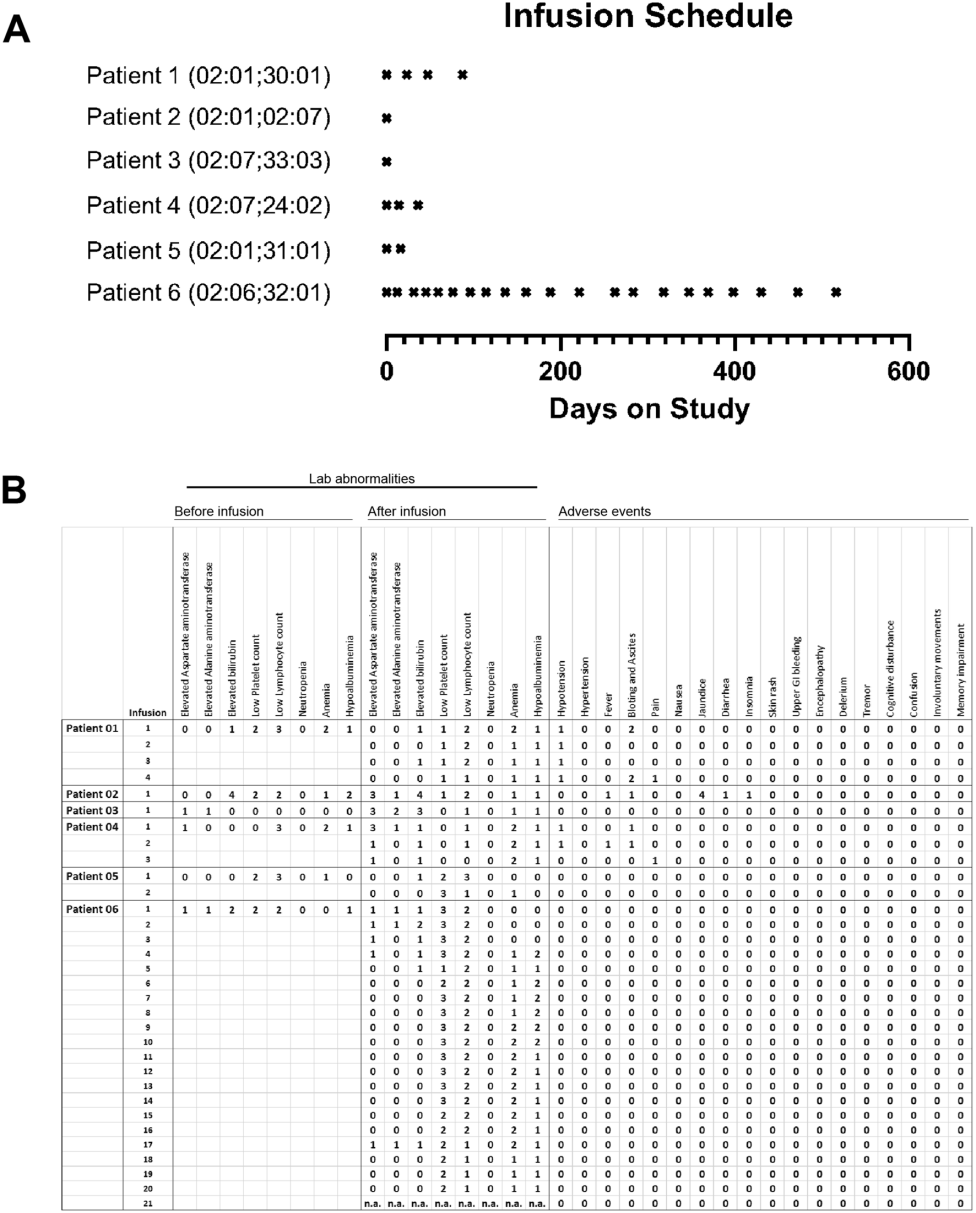
AbTCR + co-stim T cell therapy is safe in humans. (**A**) Six HCC patients with indicated HLA-A*02 genotypes were dosed as shown over a 17-month period. (**B**) Lab abnormalities were systematically recorded before the first infusion and immediately after each infusion along with adverse events. Grades are based on the CTCAE scale of 0–5; grade 0 means that physiological measurements were within normal range or that an adverse event was not observed.

We utilized the in vitro and in vivo results to gain regulatory approval for human safety trials of the AbTCR + co-stim technology. Six HLA-A*02-positive patients diagnosed with advanced hepatocellular carcinoma were enrolled in this study and infused at least once with autologous AbTCR + co-stim T cells, with total T cell dosage ranging from 210 to 2436 million (Figs. 3A, S8).

Remarkably, not a single patient exhibited cytokine release syndrome and treatment-related adverse events were rare and mild (Fig. 3B). As such, immunosuppressive medications such as prednisone and tocilizumab were not prescribed. Two patients reported a mild fever that resolved upon acetaminophen administration. The most common lab abnormality was a slightly reduced lymphocyte count, and low platelets were also occasionally reported in Patient 6. Patient 2 exhibited jaundice, but this is most likely the result of pre-existing liver disease because elevated bilirubin was observed before infusion. Although serum levels of cytokines were not systematically collected, the available results indicate a clean safety profile for this TCRm-based cell therapy.

**Figure 4:**
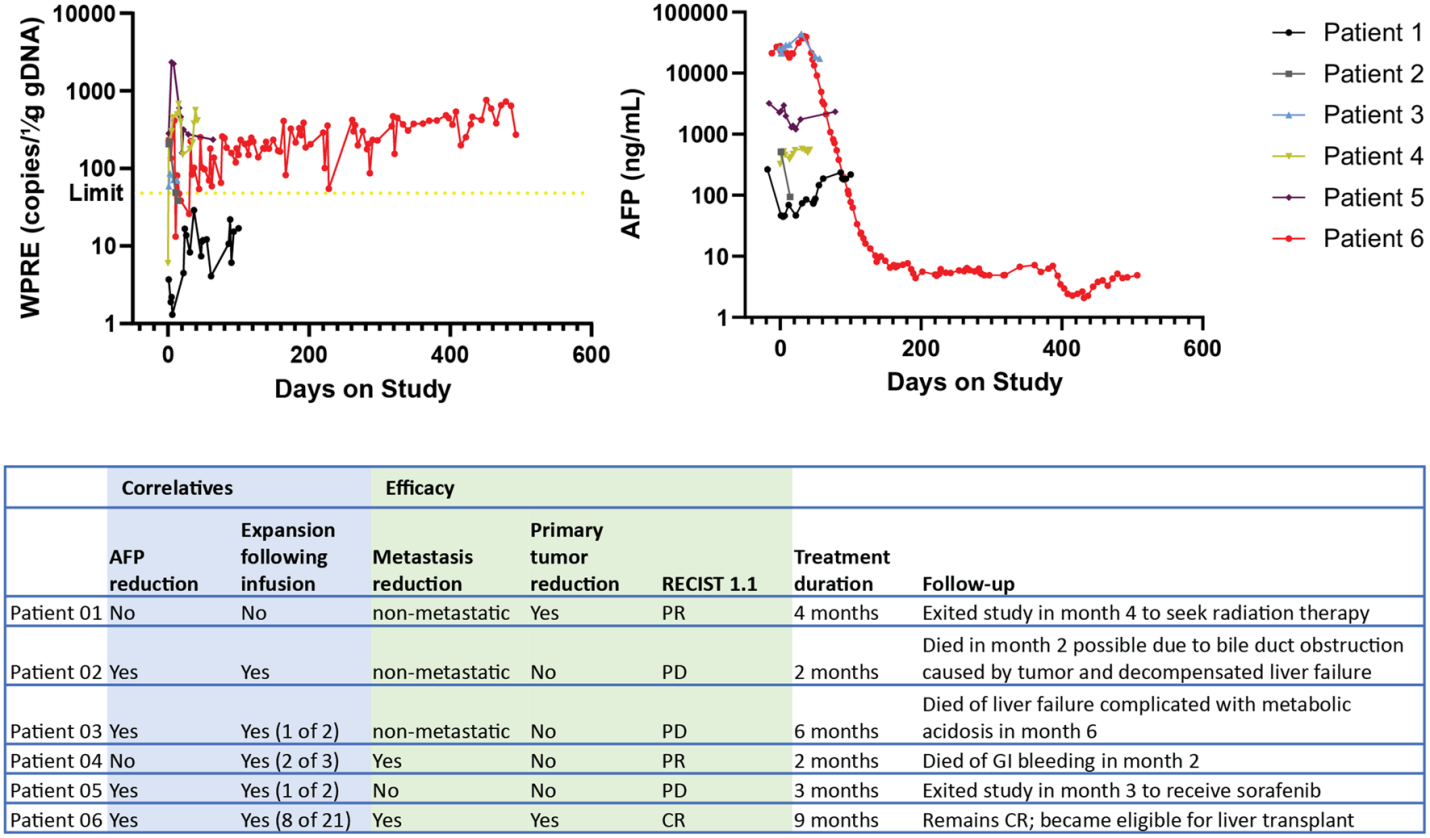
AbTCR + co-stim receptor-positive cells can expand after infusion, reduce serum AFP levels and cause HCC tumors to regress. Each patient was assayed for vector WPRE expression by PCR analysis and serum AFP level by ELISA the day after infusion. The detection limit for the PCR is shown; each value for the clinical-grade AFP ELISA is within range of the assay. The table summarizes the correlative relationships between receptor-positive T cell expansion and AFP reduction on an infusion-by-infusion basis. Efficacy is summarized in terms of regression observed and RECIST1.1 score at the conclusion of treatment.

In addition to its safety, the AbTCR + co-stim T cells exhibited evidence of activity in humans (Fig. 4). In most patients, infused T cells expanded after infusions according to WPRE copy number analysis and this was accompanied by a commensurate drop in serum AFP as shown. In Patient 6, we observed particularly significant T cell expansion and AFP decline. The positive slope in WPRE copy number in Patient 6 suggests that previously dosed T cells perdured as additional T cells were infused over time. This patient’s RECIST1.1 score was CR after nine months and their health was so improved that they ultimately qualified for a liver transplant.

**Figure 5:**
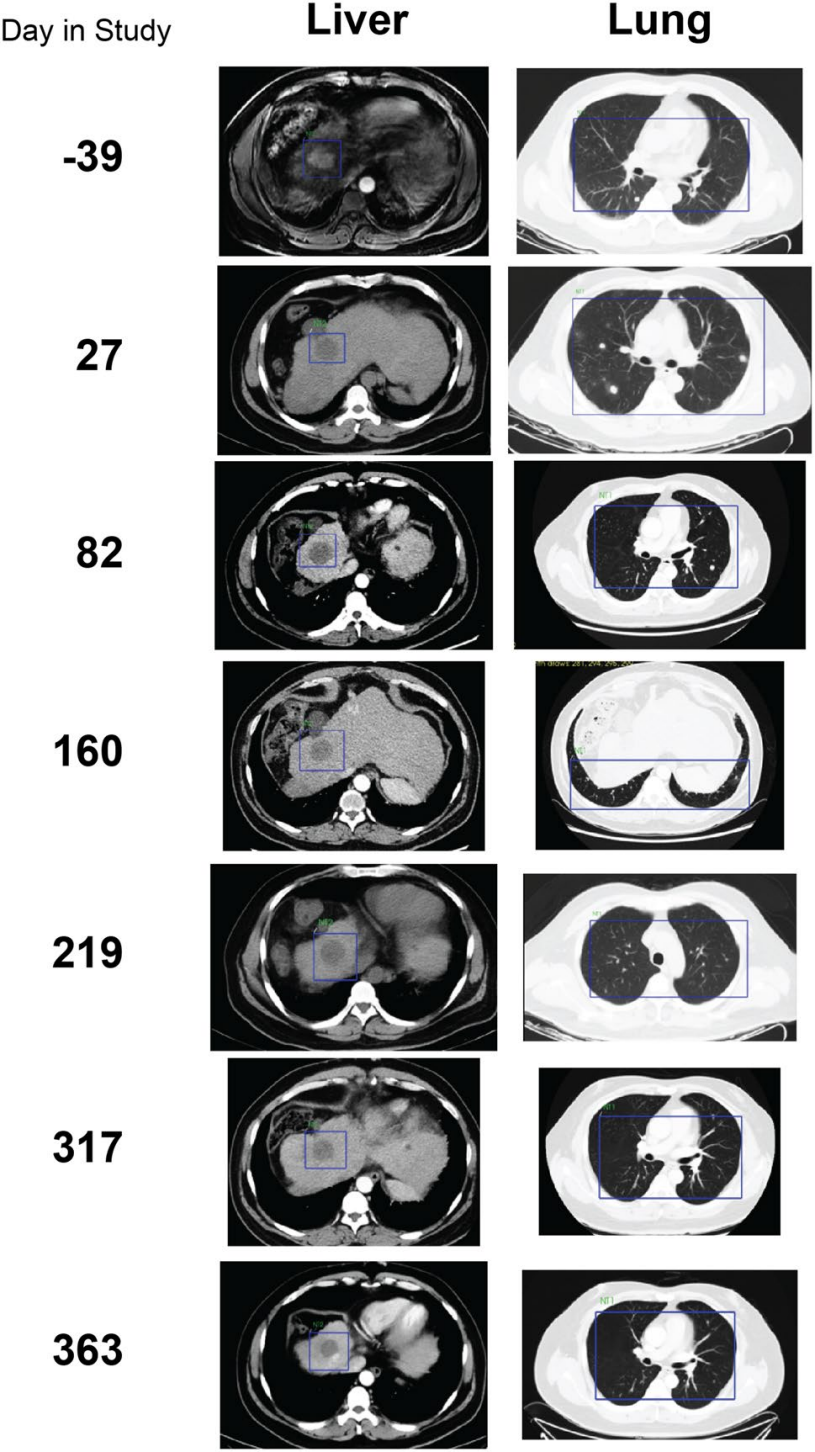
Regression of primary tumor and metastases in Patient 6. A primary tumor is visible in the liver prior to the study start (blue box, day -39) but only the tumor-free cavity remains in subsequent images throughout the course of the study. In the lung, multiple metastases present as white spots throughout the first 82 days of imaging but are undetectable by day 160 and remain so for the remainder of the study. All images are MRI with the exception of day −39 for the liver, which is CT.

We also performed imaging of primary and metastatic sites in Patient 6 (Fig. 5). Most primary tumors in the liver and all metastases in the lung responded well to the treatment. One tumor in the liver visible prior to the start of the study disappeared with treatment and remained so throughout the duration of the study. In the lung, multiple tumors were detected over the course of the study, but all resolved by day 363. These data demonstrate that the AbTCR + co-stim T cell platform is safe in humans with the potential to treat advanced metastatic HCC without serious side effects.

## Discussion

The anti-tumor activity of TCRm technology has been reported in in vitro and animal studies. TCRm antibodies have been shown to mediate antibody-dependent cytotoxicity^45–47^, engage T cells^48–51^, and function as chimeric antigen receptors^13,52^. While these studies show how well TCRm antibodies lyse cancer cells and address specificity in vitro or in animal models, they do not address the significant safety hazard of non-specific, or off-target, pMHC recognition in humans. The clinical consequences of non-specifically targeting pMHC have been well documented. In one case, an anti-MAGE TCR recognized epitopes present in other MAGE family members expressed in healthy cells, leading to fatalities^1^. A different anti-MAGE TCR recognized an unrelated peptide derived from a protein enriched in muscle; severe cardiac toxicity and death ensued^2,3^. Significant side effects have also been caused by TCRs intended to target MART-1^4^ and carcinoembryonic antigen^5,6^. Our results here show that, in contrast with these reports, a carefully constructed T cell therapy centered around a highly specific TCRm antibody does not pose a significant risk to safety and has the potential to successfully treat solid tumors.

One might hypothesize that the nanomolar affinities achievable with TCRm antibodies could reduce the off-target tendencies of T cell-based therapies. With naturally occurring TCRs, this has generally not been the case, as higher affinity has been associated with increased cross-reactivity^53–56^, likely due to the difficulty of targeting a single epitope in the context of the larger MHC protein as well as the mechanisms for cross-reactivity built into TCRs^57^. Nanomolar-binding TCRm antibodies have shown similarly limited specificities; we previously characterized a TCRm that detects a PRAME-derived epitope but could tolerate only three mutations in the peptide^47^. A related concern of nanomolar binding TCRs, and by extension TCRm antibodies, is the possible decrease in T cell signaling potency that has been associated with very high affinity binding^53,56,58,59^.

As the hazards and concerns of TCRs are shared by TCRm antibodies, the development of TCRm therapies has been significantly impaired. Our findings directly address these concerns. The TCRm in the current study is highly specific: our studies showed that 7 of the 9 residues of the AFP peptide presented by HLA-A*02 cannot be mutated without affecting binding, and in vitro and animal studies demonstrated no off-target effects^13^. Importantly, we also demonstrate here that the anti-AFP TCRm antibody in a AbTCR format, expressed along with an anti-GPC3 co-stimulatory molecule, is also specific in humans. The structural data indicates that fine specificity towards the AFP epitope emerges from the way the TCRm binds, engaging the entire length of the peptide rather than relying on a singular peptide hot spot as often seen with TCRs or other TCRm molecules. Broader specificity can be traced to how the TCRm interacts with the HLA-A*02 protein: although its binding geometry is TCR-like, naturally occurring αβ TCR genes have evolved alongside those of the MHC, leading to a genetically encoded predisposition for MHC binding and contributing to the cross-reactivity necessary for normal immune function^7–10^. The use of an antibody, rather than TCR germline-encoded loops to bind the MHC protein removes this built-in MHC bias. Indeed, we hypothesize that a reason that the AbTCR functions well despite nanomolar binding is that, unlike high affinity TCRs, high specificity is retained at high affinity, limiting T cell overstimulation via cross-reactivity.

As a way to further address broad specificity, most patients in our study expressed other HLA-A alleles in addition to HLA-A*02, suggesting that in vivo the AbTCR TCRm did not cross-recognize MHC molecules of other HLA-A alleles. We did not observe any concerning increase of side effects in these patients, in line with our pre-clinical observations, including the structural data showing direct engagement of HLA-A*02 polymorphisms. These data however also imply that this AbTCR + co-stim is unlikely to be effective in patients who lack HLA-A*02 expression. Additionally, HLA-A*02 dysregulation is a potential resistance mechanism. While this is often a concern in immunotherapy^60^, the AbTCR approach is independent of other mechanisms of resistance (such as BRAF mutations in HCC, which can lead to resistance against tyrosine kinase inhibitors^61^) and could thus be beneficial when used supplemental to or along with other therapies.

Another concern is that repeated exposure to AbTCR + co-stim through multiple infusions could increase the risk of side effects. However, the patient who received the largest dose of AbTCR + co-stim T cells (> 2 million T cells, Fig. S8) over the longest period experienced the greatest benefit, without serious treatment-related adverse events. In fact, his overall health improved so much that he became eligible for and ultimately received a liver transplant.

We are now enrolling patients in multi-center United States trials (NCT04502082, NCT04634357) for a T cell therapy targeting AFP and GPC3 with the identical antibody domains validated in the current study. These trials will further our understanding of TCRm safety and efficacy and provide HCC patients access to a potential cure.

## Supplementary Information

Supplementary Information.

## Data Availability

Crystallographic data have been submitted to the Protein Data Bank (https://www.rcsb.org/) and are available under accession codes 7RE7, 7RE8, and 7RE9.
