# Supplementary material for: Validation and promise of a TCR mimic antibody for cancer immunotherapy of hepatocellular carcinoma
Source: Sci Rep. 2022 Jul 15;12:12068. doi: 10.1038/s41598-022-15946-5 (PMC9287321; doi:10.1038/s41598-022-15946-5)
Supplement: Supplementary file 1 — Supplementary Information. [file 41598_2022_15946_MOESM1_ESM.docx]

**Supplementary Data**

Validation and promise of a TCR mimic antibody for cancer immunotherapy of hepatocellular carcinoma

Chang Liu, Hong Liu, Moumita Dasgupta, Lance M. Hellman, Xiaogang Zhang, Kai Qu, Hui Xue, Yun Wang, Fenling Fan, Qi Chang, Duo Yu, Linhu Ge, Yu Zhang, Ziyou Cui, Pengbo Zhang, Bradley Heller, Hongbing Zhang, Bingyin Shi, Brian M. Baker, and Cheng Liu

Fig. S1. AbTCR+co-stim can form a signaling-competent cytolytic synapse.

Fig. S2. The co-stim confers a level of cytokine release, T cell proliferation and central memory compartment expansion on par with CD80.

Fig. S3. T cells co-expressing AbTCR and co-stim but not co-stim alone release cytokines and degranulate in the presence of AFP+GPC3+ target cells.

Fig. S4. Electron densities for key regions in the crystallographic structures.

Fig. S5. Interatomic contacts in the TCRm-AFP/HLA-A*02 interface.

Fig. S6. SPR sensorgram for the TCRm binding AFP/HLA-A*02.

Fig. S7. Comparison of free and bound TCRm and AFP/HLA-A*02.

Fig. S8. Characteristics of HCC patients enrolled in study.

Table S1. X-ray data and refinement statistics.


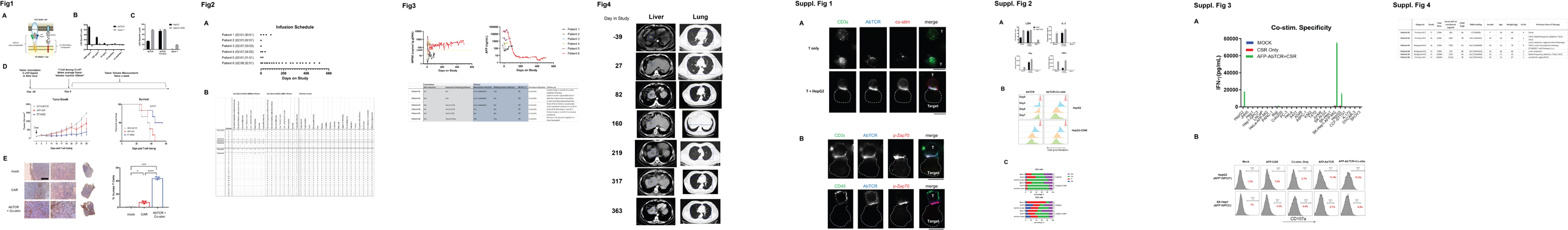


Fig. S1. AbTCR+co-stim can form a signaling-competent cytolytic synapse. (A) AbTCR and co-stim receptors in T cells were co-stained with CD3ε before target engagement (top row) or after engagement with HepG2 cells (bottom row). An un-transduced CD3ε + T cell is shown in the top of the field to demonstrate specificity of staining. Upon target engagement by the AbTCR, co-stim and CD3ε co-localize at the presumptive synapse. (B) AFP-AbTCR+co-stim T cells were co-cultured with SK-HEP-1 cells (ATCC#: HTB-52) engineered to express the AFP peptide and stained for canonical signaling molecules of the immune synapse. Phosphorylated Zap70 co-localizes with AbTCR and CD3ε at the interface of the T cell and target cell (top row). In a distinct example (bottom row), CD45 is mostly excluded from this region as expected. Scale bar 10 µm.


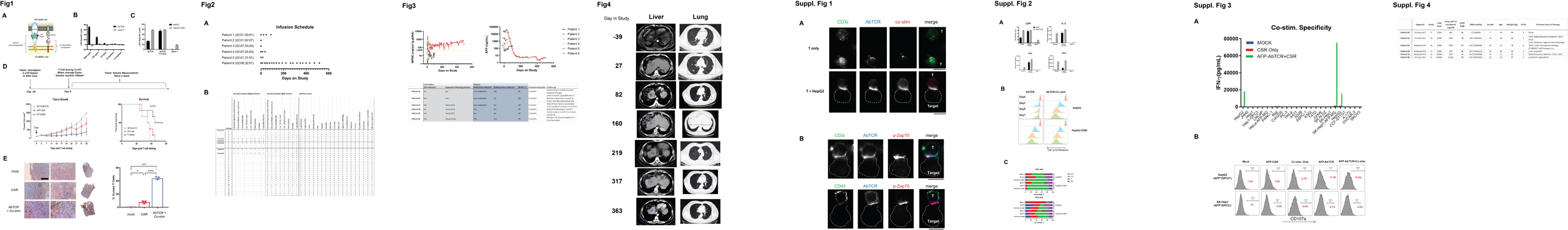


Fig. S2. The co-stim confers a level of cytokine release, T cell proliferation and central memory compartment expansion on par with CD80. (A) *In vitro* cytotoxicity of T cells was measured by a LDH release assay to quantify specific lysis of target cells. Concentrations of secreted cytokines in supernatants were quantified after 16 hours of incubation. (B) T cells were labeled with the CFSE dye and co-cultured with HepG2 cells, and the degree of T cell proliferation was assessed by flow cytometry. After 7 days of co-culturing, the remaining T cells were rechallenged with fresh target cells for another 3 days and analyzed by flow cytometry to assess T cell subsets (C). Frequency of T_naïve_ (CCR7+ CD45RA+), central memory (T_cm_; CCR7+ CD45RA−), effector memory (T_em_; CCR7- CD45RA-) and effector (T_eff_; CCR7- CD45RA+) T cells within CD8+ receptor+ cells. Error bars: SEM. Representative results from 3 donors are shown for each assay.

Fig. S3. T cells co-expressing AbTCR and co-stim but not co-stim alone release cytokines and degranulate in the presence of AFP+GPC3+ target cells (A) AFP-AbTCR+co-stim. T cells, Mock (un-transduced) T cells, or co-stim alone. T cells were co-incubated at an E:T ratio of 1:1 with tumor cells from various tissues. At 16 hours, the concentration of IFN-γ was quantified in supernatants with Luminex Magpix technology using BioRad Bio-Plex kits. HeLa-MG cells are engineered to express the AFP158 peptide region (AFP mini-gene, MG). (B) AFP-AbTCR+co-stim. T cells, AFP-AbTCR T cells, Mock T cells, or co-stim alone. T cells were co-incubated with Hep G2 (AFP+/GPC3+) or SK-HEP-1 (AFP-/GPC3) cells for 4 hours. The accumulation of CD107a was determined by flow cytometry as a measurement of cellular degranulation.

**Fig. S4.** **Electron densities for key regions in the crystallographic structures.** (A) Density for the CDR loops and the peptide in the TCRm-AFP/HLA-A*02 structure. (B) Density for the peptide in the free AFP/HLA-A*02 structure. (C) Density for the CDR loops in the free TCRm Fab. For panels a, b, and c, densities are 2F_o_-F_c_ contoured at 1σ. (D) 2F_o_-F_c_ composite OMIT map at 1σ for the peptide in the ternary complex structure. For all panels, densities are for the first molecules in each asymmetric unit as indicated in the Methods.


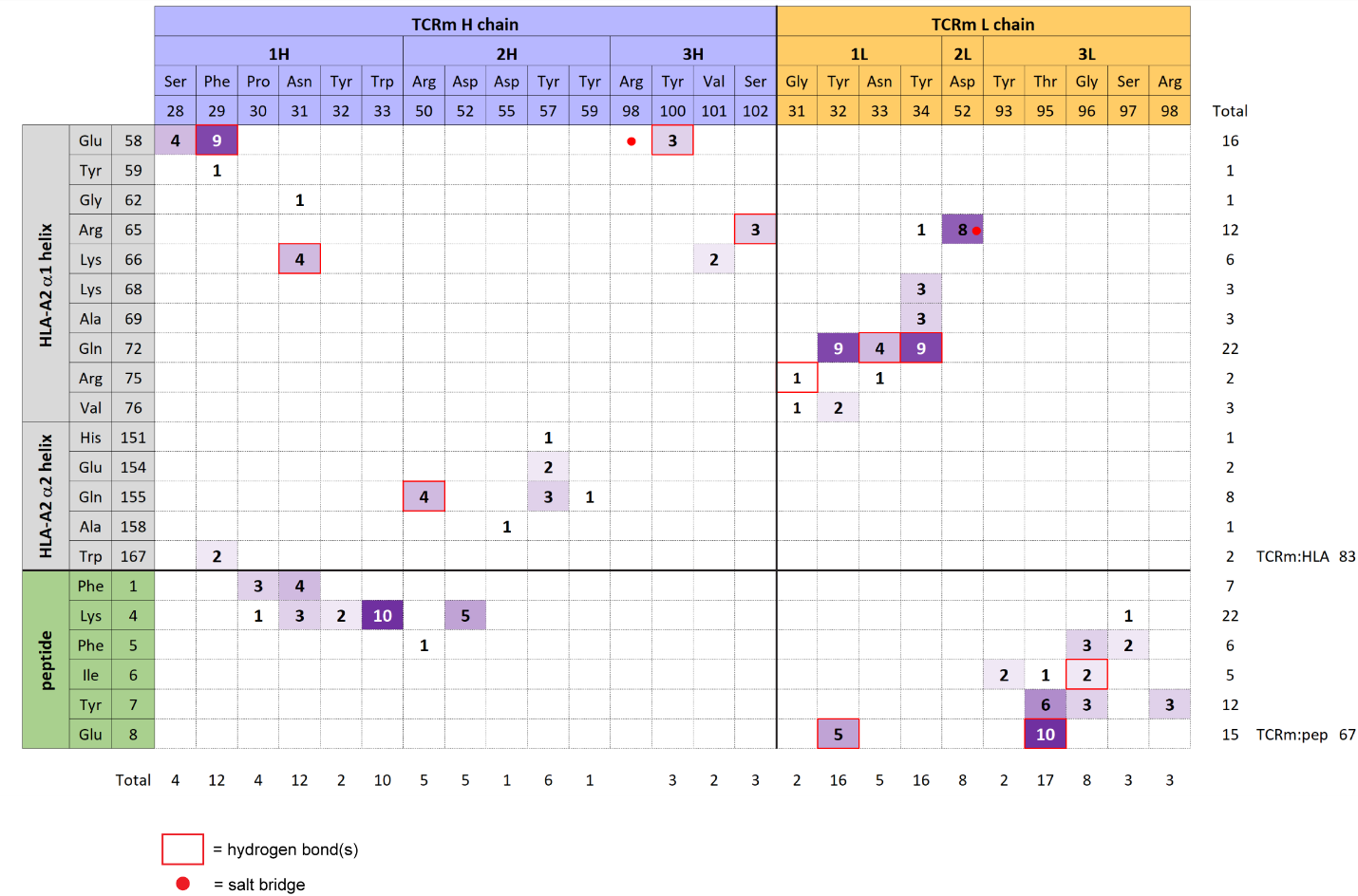


**Fig. S5.** **Interatomic contacts in the TCRm-AFP/HLA-A*02 interface.** Cells are colored according to the number of contacts from white (minimum) to purple (maximum). Contacts are defined as interatomic distances ≤ 4 Å. The presence of a hydrogen bond is indicated by a red outline. The presence of a salt bridge is indicated by a red dot (salt bridges can be > 4 Å).


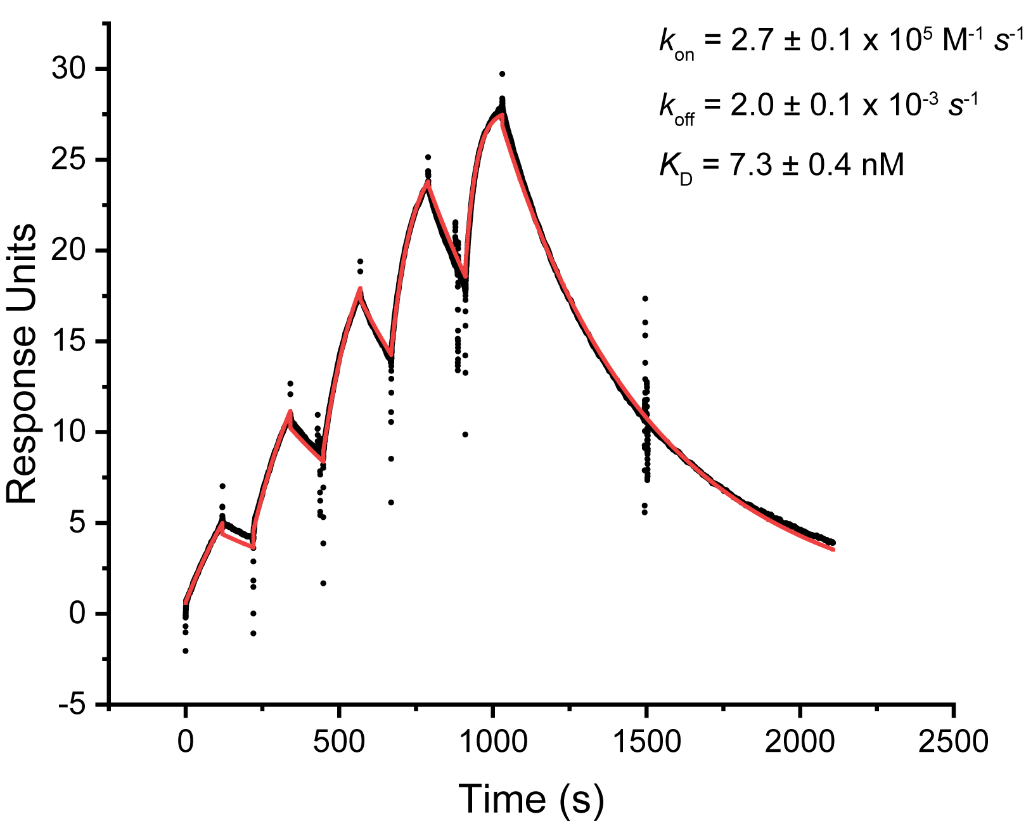


**Fig. S6. SPR sensorgram for the TCRm binding AFP/HLA-A*02** The data show a kinetic titration, with data points in black and a fit to a 1:1 binding model in red. The sensorgram is representative of three experiments. The *k*_on_, *k*_off_, and *K*_D_ are shown in the inset and are the means and standard deviations of the three measurements. The TCRm binds with a high affinity of 7 nM, determined by a fast association rate and a relatively slow dissociation rate.

B

A

**Fig. S7. Comparison of free and bound TCRm and AFP/HLA-A*02** (A) The overall conformation of the AFP peptide is unchanged upon TCRm binding except for changes in side chain rotamers at positions 4 and 5. The TCRm-free and TCRm-bound peptides superimpose with an RMS deviation of 0.4 Å for Cα atoms and 1.7 Å for all atoms. (B) The TCRm undergoes slight conformational changes upon binding, most notably in rigid body shifts in the positioning of VL relative to VH that leads to alterations in the positions of the CDR loops. The free and bound variable domains of the TCRm superimpose with an RMS deviation of 1.4 Å for Cα atoms and 1.9 Å for all atoms. For both panels, analyses are for the first molecules in each asymmetric unit as indicated in the Methods.

**
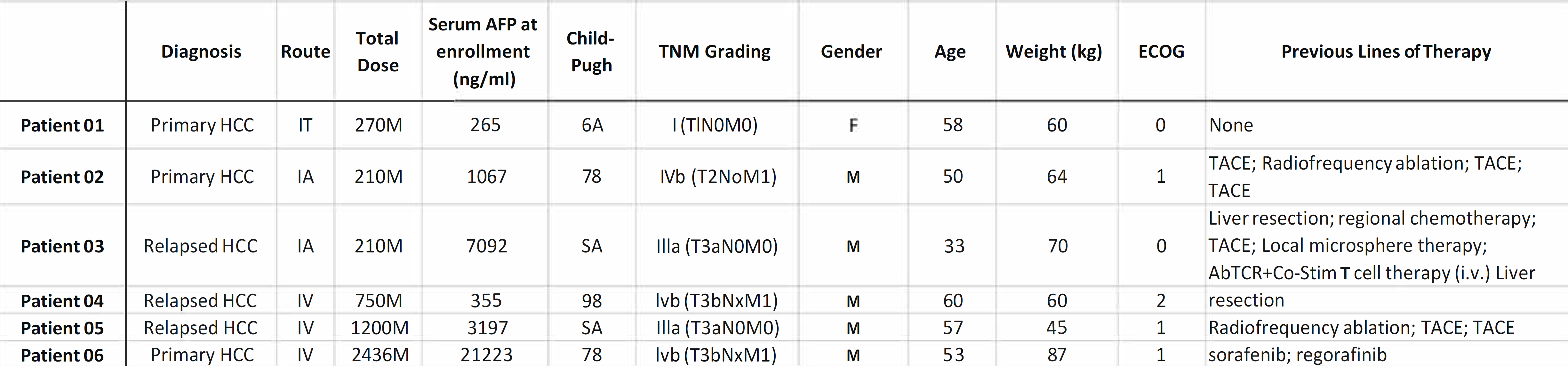
**

**Fig. S8. Characteristics of HCC patients enrolled in study** Among the patients, half were primary HCC and half were relapsed. All but the least advanced patient experienced previous lines of therapy. Serum AFP, Child-Pugh and ECOG scores are shown for each patient at the start of the study.

**Table S1.** X-ray data and refinement statistics

^[*]^ Values in parentheses are for highest resolution shell.

|  | **TCRm-AFP/HLA-A*02** | **Free AFP/HLA-A*02** | **Free TCRm** |
| --- | --- | --- | --- |
| **Data Collection** |  |  |  |
| Resolution range (Å) ^[*]^ | 48.25 - 2.55 (2.64 - 2.55) | 58.47 - 2.82 (2.92 - 2.82) | 66.33 - 2.77 (2.87 - 2.77) |
| Space group | P 21 21 21 | P 1 21 1 | C 1 2 1 |
| Unit cell dimensions (Å) | 49.7, 200.9, 244.6 | 63.2, 87.3, 78.7 | 134.3, 40.4, 161.8 |
| Unit cell angles (°) | 90, 90, 90 | 90, 90, 90 | 90, 105.2, 90 |
| Unique reflections | 81128 (7652) | 20564 (1911) | 21759 (2197) |
| Completeness (%) | 96.4 (85.0) | 97.2 (93.9) | 97.8 (96.3) |
| Mean I/sigma(I) | 19.9 (1.7) | 8.4 (1.5) | 8.9 (1.6) |
| R-merge | 0.17 (0.87) | 0.15 (0.63) | 0.34 (1.81) |
| R-meas | 0.18 (0.92) | 0.17 (0.69) | 0.38 (1.97) |
| R-pim | 0.00 (0.30) | 0.06 (0.26) | 0.15 (0.77) |
| CC1/2 | 0.99 (0.76) | 0.99 (0.90) | 0.97 (0.56) |
|  |  |  |  |
| **Refinement** |  |  |  |
| Reflections used in refinement | 78753 (6845) | 20182 (1912) | 21483 (2120) |
| Reflections used for R-free | 7854 (674) | 2019 (194) | 2147 (214) |
| R-work | 0.19 (0.26) | 0.19 (0.24) | 0.23 (0.32) |
| R-free | 0.22 (0.31) | 0.23 (0.29) | 0.26 (0.38) |
| Wilson B-factor | 37.5 | 28.2 | 27.5 |
| Number of non-hydrogen atoms | 13099 | 6558 | 6377 |
| macromolecules | 12743 | 6338 | 6309 |
| ligands | 197 | 162 | 14 |
| solvent | 159 | 58 | 54 |
| Protein residues | 1619 | 768 | 840 |
| RMS (bonds) | 0.005 | 0.008 | 0.011 |
| RMS (angles) | 0.72 | 1.3 | 1.45 |
| Ramachandran favored (%) | 97.9 | 97.5 | 97.0 |
| Ramachandran allowed (%) | 2.1 | 2.5 | 3.0 |
| Ramachandran outliers (%) | 0.06 | 0 | 0 |
| Rotamer outliers (%) | 0.4 | 2.1 | 4.2 |
| Clashscore | 3.7 | 6.0 | 3.0 |
| \| PDB ID Code \| \| --- \| | 7RE7 | 7RE8 | 7RE9 |
